# MRI load of cerebral microvascular lesions and neurodegeneration, cognitive decline, and dementia

**DOI:** 10.1212/WNL.0000000000006355

**Published:** 2018-10-16

**Authors:** Rui Wang, Anna Laveskog, Erika J. Laukka, Grégoria Kalpouzos, Lars Bäckman, Laura Fratiglioni, Chengxuan Qiu

**Affiliations:** From the Department of Neurobiology (R.W., E.J.L., G.K., L.B., L.F., C.Q.), Care Sciences and Society, Aging Research Center, Karolinska Institutet and Stockholm University; Division of Radiology (A.L.), Department of Clinical Science, Intervention and Technology, Karolinska University Hospital at Huddinge; Department of Neuroradiology (A.L.), Karolinska University Hospital, Stockholm; and Stockholm Gerontology Research Center (L.F.), Stockholm, Sweden.

## Abstract

**Objective:**

To explore the differential associations of neurodegeneration and microvascular lesion load with cognitive decline and dementia in older people and the modifying effect of the *APOE* genotype on these associations.

**Methods:**

A sample of 436 participants (age ≥ 60 years) was derived from the population-based Swedish National study on Aging and Care in Kungsholmen, Stockholm, and clinically examined at baseline (2001–2003) and 3 occasions during the 9-year follow-up. At baseline, we assessed microvascular lesion load using a summary score for MRI markers of lacunes, white matter hyperintensities (WMHs), and perivascular spaces and neurodegeneration load for markers of enlarged ventricles, smaller hippocampus, and smaller gray matter. We assessed cognitive function using the Mini-Mental State Examination (MMSE) test and diagnosed dementia following the *Diagnostic and Statistical Manual of Mental Disorders*, 4th edition criteria. We analyzed data using linear mixed-effects, mediation, and random-effects Cox models.

**Results:**

During the follow-up, 46 participants were diagnosed with dementia. Per 1-point increase in microvascular lesion and neurodegeneration score (range 0–3) was associated with multiple adjusted β-coefficients of −0.35 (95% confidence interval, −0.51 to −0.20) and −0.44 (−0.56 to −0.32), respectively, for the MMSE score and multiple adjusted hazard ratios of 1.68 (1.12–2.51) and 2.35 (1.58–3.52), respectively, for dementia; carrying *APOE* ε4 reinforced the associations with MMSE decline. WMH volume changes during the follow-up mediated 66.9% and 12.7% of the total association of MMSE decline with the baseline microvascular score and neurodegeneration score, respectively.

**Conclusions:**

Both cerebral microvascular lesion and neurodegeneration loads are strongly associated with cognitive decline and dementia. The cognitive decline due to microvascular lesions is exacerbated by *APOE* ε4 and is largely attributed to progression and development of microvascular lesions.

Cerebral small vessel disease (SVD) is visualized on MRI as white matter hyperintensities (WMHs), lacunes, perivascular spaces (PVSs), microbleeds, and brain atrophy.^1^ Studies have shown that SVD plays a pivotal role in cognitive decline and dementia,^2–4^ but most studies have examined individual markers. Because SVD markers often occur concurrently in older people, a cluster of SVD markers may better capture the extent and severity of microvascular and neurodegenerative damage than single markers and better reflect their cognitive phenotypes.^5^ Cross-sectional studies have suggested a strong correlation of heavy SVD load with cognitive deficits,^6,7^ whereas longitudinal data are still lacking.

Furthermore, neuropathologic studies have correlated MRI markers of regional and global brain atrophy (e.g., hippocampal atrophy and enlarged ventricles) with neurodegenerative pathologies.^8,9^ Notably, neurodegenerative and microvascular pathologies share common mechanisms (e.g., inflammation and oxidative stress) and have a reciprocal relationship. For instance, cerebral arteriosclerosis and hypoxia impair perivascular drainage of amyloid and increase neurodegeneration, whereas Alzheimer-related pathologies cause auxiliary vascular damage.^10–12^ Thus, SVD or atrophic markers are likely to reflect a mixture of microvascular and neurodegenerative pathology in the brain.

Therefore, several issues on SVD load and cognitive phenotypes remain to be clarified: (1) whether various SVD markers, which represent different dominant pathologies such as intrinsic arteriolar disease (lacunes and WMH) and neurodegeneration (hippocampal atrophy), have differential effects on cognition is unclear; (2) the extent to which the association of SVD load with cognitive phenotypes is attributed to subsequent structural brain changes is unknown; and (3) whether *APOE* ε4 interacts with SVD load to affect cognitive decline and dementia has yet to be determined. In this population-based cohort study, we seek to assess the differential associations of microvascular lesion and neurodegeneration load with cognitive decline and dementia and to examine the effect of *APOE* ε4 on these relationships.

## Methods

### Study participants

**Figure 1:**
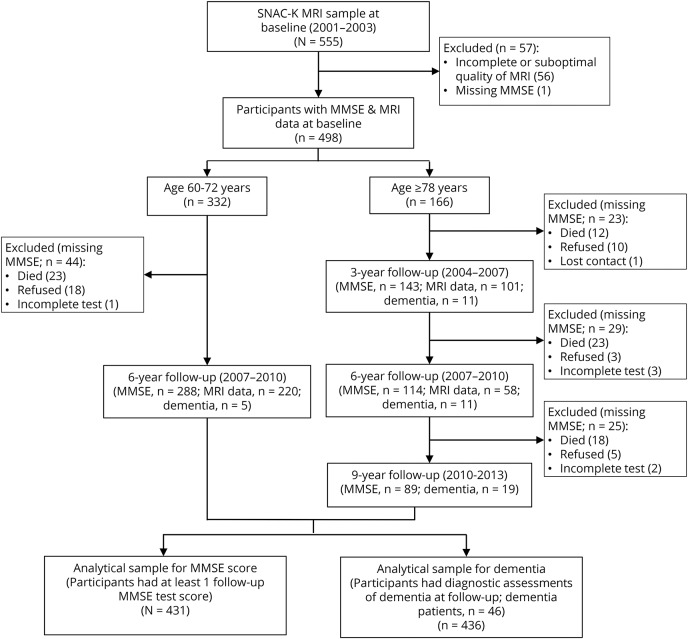
Flowchart of the study participants in SNAC-K MRI, 2001–2003 to 2010–2013 MMSE = Mini-Mental State Examination; SNAC-K = Swedish National study on Aging and Care in Kungsholmen.

Participants in this population-based cohort study were derived from the Swedish National study on Aging and Care in Kungsholmen (SNAC-K), a multidisciplinary study of aging and health.^13^ Briefly, the SNAC-K participants consisted of an age-stratified random sample of people who were aged 60 years or older and living either at home or in institutions in the Kungsholmen district of central Stockholm, Sweden. The sample included 3 younger age cohorts, each with a 6-year interval (60, 66, and 72 years), that were followed up with every 6 years, and 8 older age cohorts, each with a 3-year interval (78, 81, 84, 87, 90, 93, 96, and 99 + years), that were longitudinally assessed every 3 years. At baseline (March 2001 to August 2004), 3,363 of the 4,590 eligible participants (73.3%) were examined. Participants of the SNAC-K MRI study represented a subsample (n = 555) of the SNAC-K participants who were noninstitutionalized and free of disability and dementia and were recruited between September 2001 and October 2003.^14,15^ In this study, we used 9-year follow-up data for the SNAC-K MRI sample (2001–2003 through 2010–2013). Of the 555 participants, 57 were excluded because of incomplete MRI data or suboptimal image quality (n = 56) or missing data on the Mini-Mental State Examination (MMSE) test at baseline (n = 1). Of the remaining 498 participants, the 431 participants who had at least 1 cognitive assessment at follow-up were included in the analysis concerning cognitive decline, and the 436 participants who had a diagnostic assessment for dementia at follow-up were included in the analysis involving dementia. In addition, 321 of the 498 participants (64.5%) had at least 1 follow-up MRI scan. Figure 1 shows the flowchart of the study participants. Compared with participants who did not undergo brain MRI, the SNAC-K MRI participants were younger and more educated, and the 2 groups did not differ in sex and *APOE* ε4 allele distribution, as previously reported.^15^

### Standard protocol approvals, registrations, and patient consents

The Ethics Committee at the Karolinska Institutet or the Regional Ethics Review Board in Stockholm, Sweden, approved all parts of the SNAC-K study, including linkage with patient register and death certificates. All participants provided written informed consent.

### MRI acquisition and reading protocol

At baseline, participants were scanned on a Philips Intera 1.5T MRI system (Eindhoven, The Netherlands).^14,15^ The core MRI protocol included an axial 3D T1-weighted fast-field-echo sequence (time of repetition [TR] 15 ms, time to echo [TE] 7 ms, flip angle [FA] 15°, field of view [FOV] 20, matrix 256 × 256), a fluid-attenuated inversion recovery [FLAIR] sequence (TR 6,000 ms, TE 100 ms, inversion time 1,900 ms, FA 90°, echo train length 21, FOV 184 × 230, matrix 204 × 256), and a proton density/T2-weighted fast spin-echo sequence (TR 4,000 ms, TE 18/90 ms, FA 90°, echo train length 6, FOV 187.5 × 250, matrix 192 × 256, 5 mm slices, without the use of gap and angulation).^14,15^

A single rater (G.K.) manually drew global WMH volume on FLAIR images and further interpolated on the corresponding T1 images to compensate for the gap between slices in FLAIR (Dice coefficients ranged 0.74–0.78, and the mean Dice coefficient was 0.76), as previously reported.^16^ We segmented T1 images into gray matter, white matter, and CSF using SPM12 in MATLAB R2012b. A specialist in neuroimaging analysis (G.K.) visually inspected all segments. Hippocampal volume in both hemispheres was manually delineated using the region of interest tool, and the lateral ventricular volumes were estimated using a region-growing-based semiautomatic tool in HERMES MultiModality.^14^ We also used automated FreeSurfer segmentation to measure hippocampal volume at baseline.^17^ The manually and automatically measured hippocampal volumes were highly correlated (r = 0.862, *p* < 0.001). In this study, we used the manual measurement of hippocampal volume, as we used in previous studies.^14,15^ We adjusted all volumetric measurements by total intracranial volume.

We defined lacunes as small lesions with CSF signal on all sequences and surrounding high signals on FLAIR sequence. We used a visual rating scale to evaluate PVS, as fully reported elsewhere.^18^ Briefly, we used T1 and T2 images to assess PVS in different brain areas (e.g., frontal lobe, parieto-occipital lobe, basal ganglia, thalami, and cerebellum) in each brain hemisphere. In each region, the number of visible PVS was measured on a 0–3 point scale: 0, no visible PVS; 1, 1–5 PVS; 2, 6–10 PVS; or 3, >10 PVS. Then, we summed up the point from all brain regions to obtain a global PVS score. A clinical neuroradiologist (A.L.) made all the assessments of PVS. The visual rating scale for PVS had the weighted κ statistic of 0.77 for both intrarater and inter-rater reliability.^18^

### Load of MRI markers for microvascular lesions and neurodegeneration

We assessed the cerebral microvascular lesion load and neurodegeneration load separately with 2 MRI scores: (1) the microvascular lesion score summarized the MRI markers of lacunes, WMH, and PVSs because these MRI markers are assumed to reflect predominantly microvascular damage in the brain^19,20^ and (2) the neurodegeneration score counted the MRI markers of enlarged ventricles, smaller hippocampus, and smaller total gray matter because these MRI markers are usually indicative of Alzheimer-related pathology or neurodegeneration.^8,9,20^ We calculated each of the 2 MRI scores by summing up the total points of the corresponding MRI markers that were concurrently present in a participant. We assigned 1 point to each of the 6 MRI markers as follows: (1) presence of lacunes, being in the fourth quartile of (2) WMH volume, (3) global PVS score, and (4) lateral ventricular volume, and being in the first quartile of (5) hippocampal volume, and (6) total gray matter volume. Both the microvascular lesion score and neurodegeneration score ranged 0–3.

### Global cognitive function and dementia

We used the MMSE test to assess global cognitive function at baseline (2001–2003) for all participants; at 3-year (2004–2007), 6-year (2007–2010), and 9-year (2010–2013) follow-ups for participants aged ≥78 years; at 6-year and 9-year follow-ups for participants aged 72 years; and at 6-year follow-up for participants aged 60 and 66 years.

We used the *Diagnostic and Statistical Manual of Mental Disorders*, 4th edition, criteria to define dementia according to a validated 3-step diagnostic procedure.^13,21^ Briefly, the examining physician made a first preliminary diagnosis of dementia based on interviews, clinical examination, and cognitive testing; then, a reviewing physician independently made a second preliminary diagnosis; in case of disagreement between the 2 preliminary diagnoses, a third opinion from a senior physician was sought to make the final diagnosis. We defined Alzheimer disease according to the National Institute of Neurological and Communicative Disorders and Stroke and the Alzheimer's Disease and Related Disorders Association criteria. For participants who had died before the subsequent follow-up examination, physicians thoroughly reviewed medical records and death certificates to determine whether the participant died with dementia or Alzheimer disease.

### Covariates

We collected data on potential confounding factors (as covariates in the analysis) through face-to-face interviews, clinical examinations, and laboratory tests. The confounding factors included demographics (e.g., age, sex, and education), lifestyles (e.g., smoking, alcohol consumption, and physical activity), medical history (e.g., hypertension, diabetes, heart disease, and stroke), use of medications (e.g., antihypertensive agents, oral hypoglycemic agents, and cholesterol-lowering drugs), weight, height, blood pressure, and biomarkers (e.g., total cholesterol, HbA1c, and *APOE* genotype).^22^ Education was measured by the maximum years of formal schooling. We classified all medications according to the Anatomical Therapeutic Chemical (ATC) classification system. Body mass index (BMI) was calculated as measured weight (kg) divided by height (m) squared, and obesity was defined as a BMI ≥30 kg/m^2^. We dichotomized smoking status as never/former vs current smoking, alcohol consumption as heavy vs no or moderate alcohol intake, and physical activity as inactivity vs fitness-enhancing activity. We defined hypertension as arterial blood pressure ≥140/90 mm Hg or current use of antihypertensive drugs (ATC codes C02, C03, and C07-C09); diabetes as having a self-reported history of diabetes, use of oral hypoglycemic agents or insulin (ATC code A10), records of diabetes in patient register, or HbA1c ≥ 6.5%; and high total cholesterol as total cholesterol >6.22 mmol/L or current use of cholesterol-lowering drugs (ATC code C10).

### Statistical analysis

We compared the baseline characteristics of participants by *APOE* ε4 status using logistic regression models, adjusting for age. We used multiple linear mixed-effects models to estimate the β-coefficient and 95% confidence interval (CI) of average annual changes in the MMSE score over the follow-up period related to baseline MRI scores for cerebral microvascular lesions and neurodegeneration.^15^ The models included the baseline MRI score, follow-up time, and their interaction term. Because we were interested in the effects of brain MRI load on MMSE changes during follow-up, we reported only β-coefficient (95% CI) for the MRI score × follow-up time interaction term, which reflects additional effect of the MRI score on annual MMSE changes. We used random-effects Cox proportional hazards models to assess the association of MRI scores at baseline with incident dementia, with follow-up time as the time scale. We verified the proportional hazards assumption by plotting log-log survival curves and the Schoenfeld residual test. We tested interactions between an MRI score and *APOE* ɛ4 allele by adding the 3-way product term of the MRI score × follow-up time × *APOE* ɛ4 allele to the model. When a statistical interaction was detected, we further performed stratifying analysis to verify the direction and magnitude of the interaction. We reported the results from 4 models: model 1 was controlled for age, sex, education, cardiovascular risk factors (e.g., smoking, diabetes, and hypertension), and *APOE* ε4 allele; in model 2, we simultaneously entered the baseline MRI scores for both cerebral microvascular lesions and neurodegeneration into model 1 to assess their independent effects on cognitive outcomes; to assess the effect of structural brain changes that occurred during the follow-ups on the association of baseline MRI scores with cognitive decline and dementia, in model 3, we added follow-up WMH volume (a marker of microvascular lesions) to model 1; and in model 4, we added follow-up total gray matter volume (a marker of neurodegeneration) to model 3. Finally, for the analysis involving cognitive decline, we also performed mediation analysis to test and quantify the mediation effect of structural brain changes occurred during the follow-up on the association between baseline MRI score and MMSE decline, in which we used bootstrapping methods to estimate the 95% CI. We used Stata 14.0 for Windows (StataCorp LP, College Station, TX) for all analyses.

### Data availability

Data related to the current study are derived from the SNAC-K and SNAC-K/MRI projects (snac-k.se). Access to these anonymized SNAC-K and SNAC-K/MRI data will be available from the corresponding author (C.Q.) upon reasonable request and approval by the SNAC-K data management and maintenance committee at the Aging Research Center, Karolinska Institutet, Stockholm, Sweden.

## Results

At baseline, the mean age of the 436 participants was 70.3 years (SD = 9.1), and 61.0% were women. After controlling for age, carriers and noncarriers of the *APOE* ε4 allele did not significantly differ for all baseline characteristics examined, except the total cholesterol level where the *APOE* ε4 allele carriers had higher total cholesterol than the noncarriers (table 1). In addition, the age- and sex-adjusted partial correlation coefficient between the baseline microvascular lesion score and neurodegeneration score was 0.05 (*p* = 0.30).

**Table 1:**
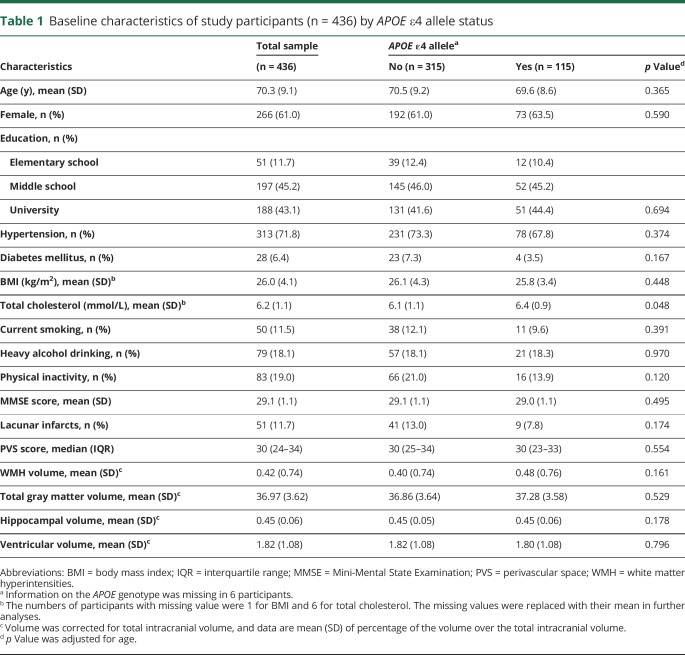
Baseline characteristics of study participants (n = 436) by *APOE* ε4 allele status

During the 9-year follow-up period, the average annual rate of MMSE changes was −0.63 (95% CI, −0.75 to −0.51; *p* < 0.001), after controlling for age, sex, and education. A higher MRI score for both microvascular lesions and neurodegeneration at baseline was associated with faster MMSE decline, independent of demographics, cardiovascular risk factors, and *APOE* ε4 status (*p* for trend < 0.01) (table 2, model 1). Furthermore, when simultaneously entering baseline MRI scores into the model, the linear association (β-coefficient) with MMSE decline remained statistically significant for both MRI scores, although the association with the neurodegeneration score appeared to be stronger than that for the microvascular lesion score (table 2, model 2).

**Table 2:**
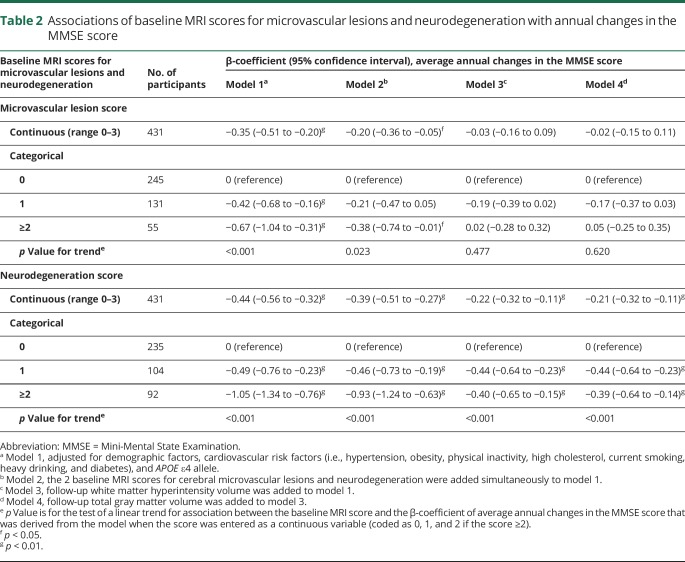
Associations of baseline MRI scores for microvascular lesions and neurodegeneration with annual changes in the MMSE score

Of the 498 baseline participants, 321 (64.5%) undertook at least 1 follow-up MRI scan, and data on follow-up WMH and total gray matter volumes were available. During the follow-up period, WMH volume increased at the average rate of 1.45 mL per year (95% CI, 1.14–1.76; *p* < 0.001) (standardized β-coefficient 0.17), whereas the total gray matter volume decreased at the average rate of 11.03 mL per year (95% CI, −12.03 to −10.03; *p* < 0.001) (standardized β-coefficient −0.15). When entering follow-up WMH volume variable into the model, the associations of both MRI scores at baseline with MMSE decline during the follow-up were substantially attenuated (table 2, model 3); notably, the linear relationship with MMSE decline remained statistically significant for the neurodegeneration score (*p* for linear trend <0.01) but not for the microvascular lesion score. Further entering follow-up total gray matter volume variable into model 3 had almost no effect on the results (table 2, model 4).

**Figure 2:**
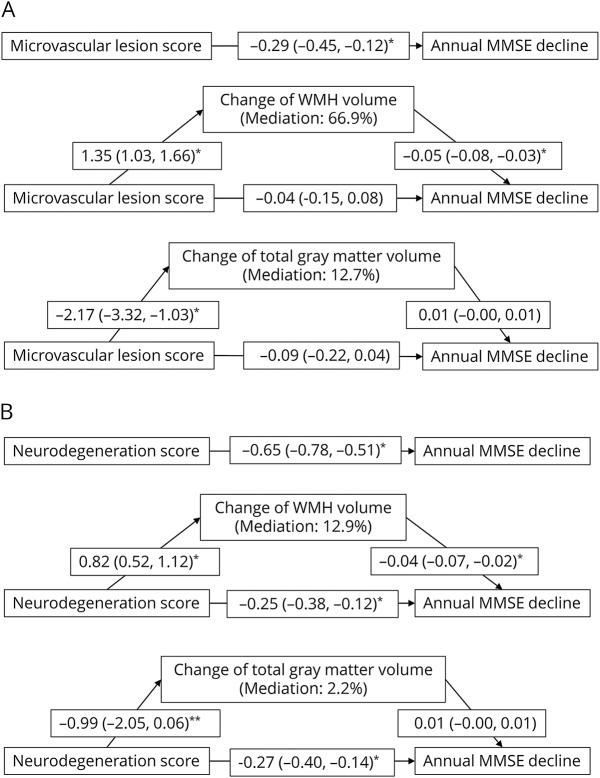
Mediation effects of changes in WMH and total gray matter volumes during the follow-up on the associations of MMSE decline with the baseline MRI score for cerebral microvascular lesions (A) and for neurodegeneration (B) Mediation models were adjusted for demographic factors, cardiovascular risk factors, and APOE ε4 allele. MMSE = Mini-Mental State Examination; WMH = white matter hyperintensities. *p < 0.01; **p = 0.066.

In the mediation analysis, after controlling for a range of potential confounders, 66.9% of the total association of MMSE decline with the baseline microvascular lesion score, but only 12.7% of the total association with the baseline neurodegeneration score, was attributed to WMH changes that occurred over the follow-up period. In contrast, changes in total gray matter volume that occurred during the follow-up period could explain only 12.8% and 2.2% of the total associations of MMSE decline with the baseline microvascular lesion score and neurodegeneration score, respectively (figure 2).

**Figure 3:**
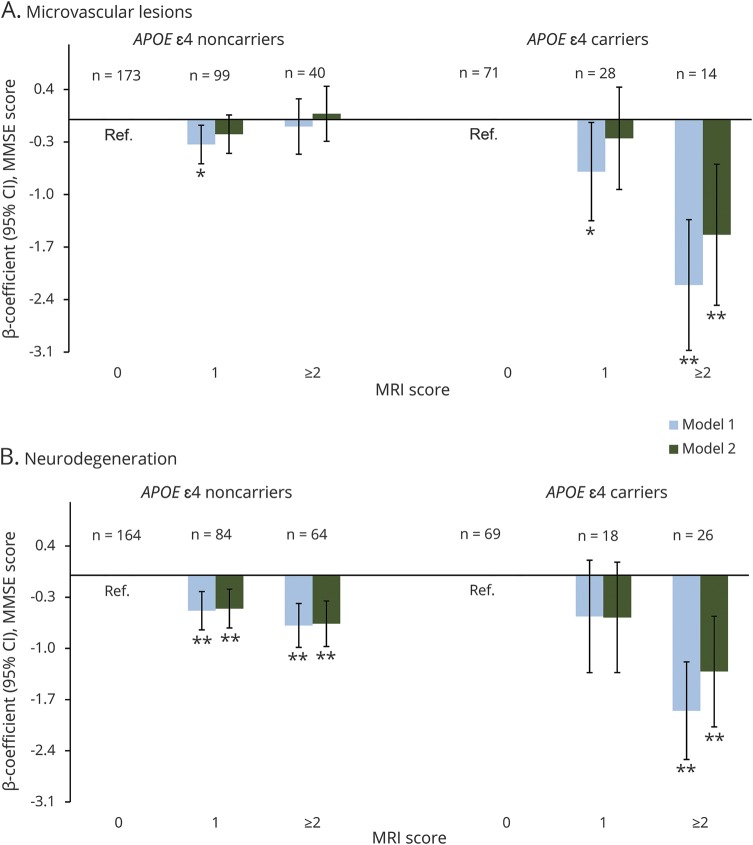
Association of the baseline MRI scores for cerebral microvascular lesions (A) and neurodegeneration (B) with average annual changes in MMSE score by *APOE* ε4 status MMSE = Mini-Mental State Examination; WMH = white-matter hyperintensities. Model 1, adjusted for demographic factors and cardiovascular risk factors; model 2, the 2 baseline MRI scores for microvascular lesions and neurodegeneration were added simultaneously to model 1. *p < 0.05; **p < 0.01.

There was a reliable interaction of *APOE* ε4 status with scores for both microvascular lesions and neurodegeneration on MMSE decline (*p* for interaction <0.01). A stratifying analysis revealed a linear association between microvascular lesions and MMSE decline only among *APOE* ε4 allele carriers (figure 3). In addition, carrying the *APOE* ε4 allele magnified the association between the neurodegeneration load and MMSE decline.

During the 9-year follow-up period, 46 participants were diagnosed with dementia; of these, 27 were classified to have Alzheimer disease. Increased MRI scores for microvascular lesions and neurodegeneration were linked to an increased risk of dementia (table 3, model 1); when simultaneously entering the 2 MRI scores into the model, the linear trend of an association with the risk of dementia was statistically significant only for the neurodegeneration score (table 3, model 2). Further adding the variables of follow-up WMH and total gray matter volumes to the model did not substantially alter the association between either score at baseline and risk of incident dementia (table 3, models 3 and 4). There was no significant interaction between the baseline MRI scores and *APOE* ε4 allele on the risk of dementia. The results for the associations between the baseline MRI scores and risk of Alzheimer disease were similar to those for dementia in general (table e-1, links.lww.com/WNL/A702).

**Table 3:**
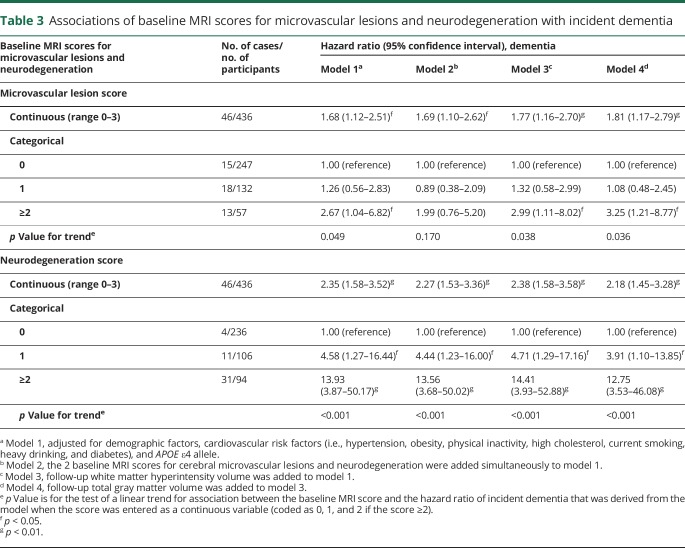
Associations of baseline MRI scores for microvascular lesions and neurodegeneration with incident dementia

Finally, we repeated the analyses only among participants who undertook at least 1 follow-up brain MRI scan and who had data available on global WMH and total gray matter volumes (n = 321), which yielded results that were consistent with those reported in tables 2 and 3 (data not shown).

## Discussion

This population-based cohort study of older adults revealed that (1) an increasing load for both cerebral microvascular lesions and neurodegeneration is strongly associated with a faster decline of global cognitive function and a greater risk of dementia and Alzheimer disease, and neurodegeneration load appears to have a stronger and more prominent association with cognitive decline and dementia risk than that of microvascular lesion load; (2) the association of microvascular lesion load with subsequent cognitive decline is largely (∼67%) attributed to the progression and development of WMH; and (3) carrying *APOE* ε4 allele strengthens the association of brain MRI load with cognitive decline, especially with regard to the cerebral microvascular lesion load.

A correlation of cerebral SVD load with global cognitive impairment and dementia has been reported in cross-sectional studies of community-dwelling older people.^6,7^ In the current cohort study, we categorized all 6 MRI markers of brain lesions into microvascular lesions and neurodegeneration and revealed a more prominent association of neurodegeneration load with cognitive decline and risk of dementia than that of microvascular lesion load. The classification of MRI markers is based on the facts that lacunes and WMH are considered to be of vascular origin because of cerebral arteriosclerosis and hypoperfusion,^19,20^ and enlarged PVS can signal microvascular lesions,^23^ whereas postmortem MRI studies correlated hippocampal atrophy with local increased burden of Alzheimer pathologies (e.g., β amyloid and tau protein) and sclerosis,^9,24^ and ventricular enlargement was a summary marker of global gray and white matter atrophy.^8,25^ However, cerebral microvascular and neurodegenerative pathologies share both risk factors (e.g., smoking, hypertension, and *APOE* gene) and biological mechanisms (e.g., inflammation and oxidative stress),^26,27^ and neurodegenerative pathologies in aging may cause cerebral perfusion deficits that precede volume loss.^28^ Also, cerebral microvascular and neurodegenerative pathologies often coexist in older people,^8,29^ and neuropathologic studies have linked volumetric brain measurements (e.g., enlarged ventricles and gray matter atrophy) with mixed Alzheimer and cerebrovascular pathologies (e.g., neuritic plaques, neurofibrillary tangles, arteriosclerosis, and infarcts).^8,20^ Thus, we should be aware that MRI markers for cerebral microvascular lesions and neurodegeneration do not necessarily represent distinct brain pathologies. Toward this end, our findings are in line with the view that the total burden of cerebral mixed vascular and neurodegenerative alterations is the strongest determinant of cognitive phenotypes in aging.^15,30–32^ The interactive effect of SVD load with *APOE* ε4 allele on cognitive decline is consistent with findings of interactions between *APOE* ε4 allele and individual markers for microvascular (e.g., WMH) and neurodegenerative (e.g., neuritic plaques and neurofibrillary tangles) lesions on cognitive phenotypes in aging.^8,10,33,34^

Both cerebral microvascular lesion and neurodegeneration loads had a strong association with subsequent cognitive decline and dementia risk in older adults. Because MRI markers for neurodegeneration are more likely to reflect mixed microvascular and neurodegenerative pathologies, the apparent stronger effect of neurodegeneration load than that of microvascular lesions on global cognitive decline and the risk of dementia suggests that clusters of various brain MRI markers with different underlying neuropathologies may act interactively to contribute to substantial cognitive decline and dementia risk.^35^ This is consistent with the clinical data showing that markers of neurodegeneration are more prominent determinants of global cognitive deficits than those of microvascular lesions (e.g., WMH, microbleeds, and lacunes).^36–38^ A cohort study of middle-aged people with a family history of Alzheimer disease also demonstrated that clusters of MRI and CSF markers for various types of brain pathology were differentially related to patterns of cognitive decline.^39^

Because nearly two-thirds of participants had follow-up MRI data in our study, we were able to explore the effect of structural brain changes on the association of baseline cerebral SVD load with cognitive decline and dementia. Our mediation analysis suggested that the association of microvascular lesion load with subsequent cognitive decline was largely mediated by follow-up changes in WMH volume, whereas the association of neurodegeneration load to cognitive decline was independent of changes in volumes of both WMH and total gray matter during the follow-up. This implies that the association between cerebral microvascular lesions and subsequent cognitive decline is attributed to the progression or new development of cerebral microvascular lesions, suggesting a vascular nature of the cognitive decline. This is a critical finding, as it may imply that interventions aiming to slow the progression and development of microvascular lesions (e.g., WMH) might attenuate cognitive decline in aging. However, we found little evidence that structural brain changes (WMH and total gray matter) influenced the link of brain MRI load to subsequent risk of dementia.

Major strengths of our study include the population-based design, longitudinal assessment of structural brain integrity, and comprehensive control of potential confounders. Our study also has limitations. First, the study sample had higher socioeconomic positions than the average Swedish population and was younger and healthier than the target population. Thus, one should be cautious in generalizing our findings to other populations. Furthermore, the MMSE test may not be sensitive to subtle cognitive changes and may be subject to practice effects when administered in a series of assessments. Third, MRI sequences for additional MRI markers for brain lesions such as cerebral microbleeds and microinfarcts were not available, which might have underestimated the effect of cerebral microvascular lesion load on cognitive outcomes. Finally, some MRI markers that we used for neurodegeneration (e.g., enlarged ventricle) may correlate also with brain vascular pathology, which needs to be kept in mind when interpreting our findings.

Overall, our population-based cohort study demonstrates a strong association of MRI load for both microvascular lesions and neurodegeneration with global cognitive decline and risk of dementia in older people. The cognitive consequences of cerebral microvascular lesion load largely stem from its progression or development of new cerebral microvascular lesions. This suggests that a structural MRI-based score approach for brain lesions may help identify individuals at risk for accelerated cognitive decline and dementia, for clinical trials, as well as for preventive interventions targeting vascular pathways that aim to delay the onset of late-life cognitive impairment and dementia.

## Publication history

Received by *Neurology* January 5, 2018. Accepted in final form July 12, 2018.

## Glossary

ATC: Anatomical Therapeutic Chemical
BMI: body mass index
FA: flip angle
FLAIR: fluid-attenuated inversion recovery
FOV: field of view
MMSE: Mini-Mental State Examination
PVS: perivascular spaces
SNAC-K: Swedish National study on Aging and Care in Kungsholmen
SVD: small vessel disease
TE: time to echo
TR: time of repetition
WMH: white matter hyperintensity
